# Skeletal Muscle Metabolic Alternation Develops Sarcopenia

**DOI:** 10.14336/AD.2021.1107

**Published:** 2022-06-01

**Authors:** Qiumei Yang, Piu Chan

**Affiliations:** ^1^Department of Neurology, Geriatrics and Neurobiology, National Clinical Research Center of Geriatric Disorders, Xuanwu Hospital of Capital Medical University, Beijing, China.; ^2^Clinical Center for Parkinson’s Disease, Capital Medical University, Beijing Institute of Geriatrics, Beijing, China.; ^3^Key Laboratory for Neurodegenerative Disease of the Ministry of Education, Beijing Key Laboratory for Parkinson’s Disease, Beijing Institute of Brain Disorders, Collaborative Innovation Center for Brain Disorders, Capital Medical University, Beijing, China.; ^4^Advanced Innovation Center for Human Brain Protection, Capital Medical University, Beijing, China.

**Keywords:** Sarcopenia, metabolic alternation, regeneration, signaling pathways

## Abstract

Sarcopenia is a new type of senile syndrome with progressive skeletal muscle mass loss with age, accompanied by decreased muscle strength and/or muscle function. Sarcopenia poses a serious threat to the health of the elderly and increases the burden of family and society. The underlying pathophysiological mechanisms of sarcopenia are still unclear. Recent studies have shown that changes of skeletal muscle metabolism are the risk factors for sarcopenia. Furthermore, the importance of the skeletal muscle metabolic microenvironment in regulating satellite cells (SCs) is gaining significant attention. Skeletal muscle metabolism has intrinsic relationship with the regulation of skeletal muscle mass and regeneration. This review is to discuss recent findings regarding skeletal muscle metabolic alternation and the development of sarcopenia, hoping to contribute better understanding and treatment of sarcopenia.

## Introduction

**Figure 1.**
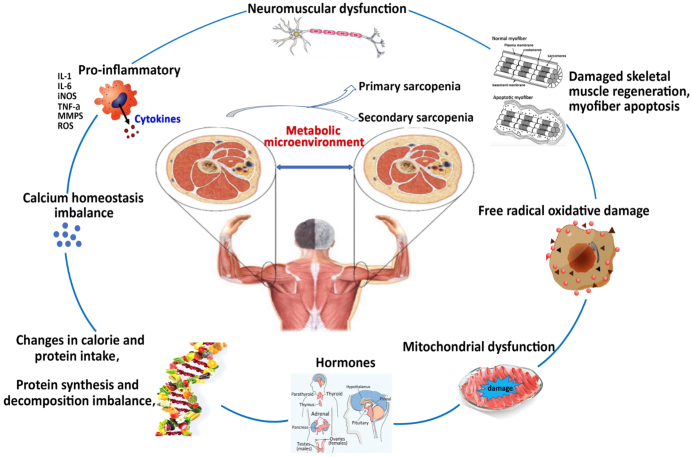
Overview of the underlying causes of sarcopenia. Decreased neuro-muscular function, Pro-inflammatory cytokines, imbalance of calcium homeostasis, imbalance in protein synthesis and decomposition, changes in caloric and protein intake, changes in hormone levels, mitochondrial dysfunction, free radical oxidative damage, skeletal muscle regeneration damage and muscle cell apoptosis. The mechanism that causes sarcopenia is the metabolic microenvironmental change in skeletal muscle.

Sarcopenia is characterized by decreased body muscle mass, muscle strength, and physical performance disorders with increasing age. Ultrastructural analysis of skeletal muscles showed that patients with sarcopenia had significant changes compared with young control, including excitatory contraction uncoupling, swelling of the transverse tube system, and sarcoplasmic reticulum fragmentation [1]. Clinically, sarcopenia is divided into two categories [2, 3]: age-related sarcopenia (also known as primary sarcopenia) has no other factors than age; secondary sarcopenia has obvious predisposing causes, such as long-term non-exercise, chronic disease, and malnutrition [4]. Sarcopenia is a complex disease that interacts with environmental and genetic factors and has multiple risk factors and develop mechanisms. Current research suggests that the cause of sarcopenia (Fig. 1) is related to decreased neuro-muscular function [5-7], pro-inflammatory cytokines [8-10], imbalance of calcium homeostasis [11], imbalance in protein synthesis and decomposition [12], changes in caloric and protein intake[13], changes in hormone levels [14-17], mitochondrial dysfunction[18-20], free radical oxidative damage [21, 22], skeletal muscle regeneration damage and muscle cell apoptosis [23]. However, the specific molecular mechanism of the onset of sarcopenia is not well understood. Recent studies have suggested that the pathological changes in sarcopenia are mainly due to changes in skeletal muscle metabolism that directly affect protein and glycogen synthesis and degradation, as well as energy utilization, leading to myofiber atrophy, skeletal muscle strength and regeneration dysfunction [24-26]. In addition, studies on primate rhesus monkeys have confirmed that changes in skeletal muscle metabolism occur before muscle dysfunction [27], suggesting that skeletal muscle metabolism changes may be one of the main causes of sarcopenia. This finding provides clues and breakthroughs for exploring the function of skeletal muscle metabolism to regulate the development and progression of sarcopenia.

## Skeletal muscle metabolism changes in sarcopenia

Skeletal muscle is composed of different metabolic types myofibers. Myofibers are classified into slow-twitch oxidized (slow-muscle), fast-twitch oxidized and fast-twitch glycolysis (fast-muscle), depending on the rate of contraction and metabolic characteristics [28]. Their metabolic microenvironment, physiological and biochemical characteristics and functions are significantly different (Table 1): Slow-twitch oxidized myofibers are characterized by a continuous extension of muscle contractility over a relatively longer time. These myofibers have a large amount of myoglobin, mitochondria, and many capillaries. This provides enough oxygen and energy to the muscles, allowing the muscles to maintain a high level of activity longer without fatigue [29, 30]. Fast-twitch oxidized myofibers are similar to slow-twitch oxidized myofibers. The difference is that the former can rapidly generate and decompose ATP through both aerobic and anaerobic metabolisms, promoting muscles with high intensity of explosiveness. Fast-twitch glycolysis myofibers contain a small amount of myoglobin, mitochondria, and capillaries, which produce ATP at a low rate through anaerobic metabolism and rapidly decompose, resulting in rapid contraction of such myofibers rapid fatigue and long-term recovery [30]. Different metabolic types of myofibers have different sensitivity to specific pathophysiological atrophy signals (Table 1). For example, slow-twitch oxidized myofibers have a higher rate of protein synthesis and degradation and are more resistant to fasting than fast-twitch glycolysis myofibers. In contrast, slow-twitch oxidized myofibers are more sensitive to inactivation, microgravity, and denervation-induced atrophy [31-34], while fast-twitch myofibers are more susceptible to cancer cachexia, diabetes, chronic heart failure, and aging [35].

**Table 1:** The characteristics of different muscle fiber types.

Fiber type	MyHC-I	MyHC-IIa	MyHC-IIx	MyHC-IIb
Activity used for	Aerobic	Long-term aerobic	Short-term anaerobic	Short-term anaerobic
Power produced	Low	Medium	High	Very high
Contraction time	Slow	Moderately fast	Fast	Very fast
Resistance to fatigue	High	Fairly high	Intermediate	Low
Maximum endurance	Hours	< 30 min	< 5 min	< 1 min
Oxidative capacity	High	High	Intermediate	Low
Glycolytic capacity	Low	High	High	High
Mitochondrial density	High	High	Intermediate	Low
Capillary density	High	Medium	Low	Low
Size of motor neuron	Small	Medium	Large	Very Large
Major storage fuel	Triglycerides	Creatine Phosphate, glycogen	Creatine phosphate, glycogen	Creatine phosphate, glycogen
Fasting tolerance	Long	Medium	Short	Short
Denervation induction atrophy sensitivity	High	Medium	Low	Low
Senescence	Slow	Very fast	Fast	Fast

**Figure 2.**
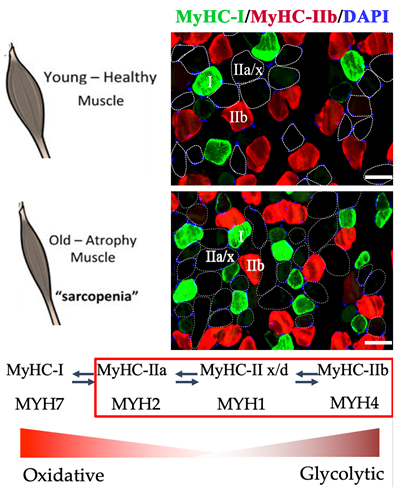
Skeletal muscle fiber type in Young-Healthy muscle and old-Atrophy muscle. Different types of skeletal muscle fibers can be converted to each other.

Some researchers have used ultrasound imaging to observe that the cross section of the quadriceps muscle in the elderly with less muscle is 25 to 35% smaller than that of the normal young people [36]. Rice et.al also found that the skeletal muscle area of ??senile sarcopenia was 28 to 36% less than that of young people, and the proportion of non-muscle tissue was significantly higher than that of young people [37]. They further identified the skeletal muscle metabolism type in patients with aging sarcopenia, and found that the fast-twitch oxidized myofibers decreased by 40% [38]. Identification of skeletal muscle metabolism types in patients with secondary sarcopenia. For example, the fast-twitch oxidative myofibers of the biceps muscle in patients with neurodegenerative diseases and long-term Parkinson's disease are reduced by up to 60% [39]; mild hypertrophic slow-twitch oxidized myofibers were identified in patients with Parkinson's muscle stiffness, whereas slow-twitch oxidized myofibers atrophy was found in muscles of Parkinson's patients with dyskinesia and mild muscle stiffness [39]. In addition, fast-twitch oxidized myofiber atrophy was also observed in sarcopenia with malnutrition and vitamin D3 deficiency: those in elderly patients were in "flat" or "fractured" shape [40]; while those in younger patients presented polygonal. The above results show that both age-related sarcopenia and secondary sarcopenia are mainly caused by fast-twitch oxidized myofibers atrophy (Fig. 2). Fast-twitch oxidized myofibers are closely related to maximum oxygen consumption, ATP production and decomposition. Therefore, the reduction of skeletal muscle oxygen consumption in patients with sarcopenia affects their demand for basic energy, leading to a decrease in energy metabolism [41], which ultimately shows a decrease in skeletal muscle strength. However, selective atrophy of myofiber types remains an important and unresolved issue.

The progressive denervation during aging have disrupted the precise overlapping between the presynaptic nerve terminal and the postsynaptic acetylcholine receptor clusters at the neuromuscular junctions (NMJ), leading to increased expression of pro-apoptotic factors and loss of nutritional factors. Neurogenic sarcopenia occurs when myofiber denervation exceeds re-innervation [42-44]. The innervated motor unit (MU) exerts a powerful influence on the metabolic phenotype of myofibers. Motor neurons are classified into fast motor neurons (F type) and slow motor neurons (S type) according to the metabolic characteristics of myofibers [45]. Under normal aging process, a preferential denervation of fast-twitch myofibers occurs and these denervated fibers are then reinnervated by the axonal sprouting from S type neurons in a process called MU remodeling, and then transformed into slow-twitch fibers [43, 44, 46]. Skeletal muscle metabolism type alternation affected the function of satellite cells (SCs). Correspondingly, degenerated NMJs were associated with reduced contribution from SCs [47]. Indeed, SCs depletion was sufficient to induce NMJ degeneration at a younger age. Conversely, prevention of SCs and derived myonuclei loss was associated with attenuation of age-related NMJ degeneration and muscle atrophy [48]. Therefore, the changes in skeletal muscle metabolism caused by re-innervation after denervation, which in turn causes the function of SCs of myofibers to decrease, may be another important factor in neurogenic sarcopenia.

## Skeletal muscle metabolism type alternation and skeletal muscle regeneration

Skeletal muscle regeneration depends on the SCs stored in the muscle. It is located between the myofiber membrane and the basement membrane and is closely related to the number and function of myofibers. The number, characteristics, and functions of SCs located on myofibers of different metabolic types are different. Recently, Dr. Zhang Yong's team proposed that the skeletal muscle fiber metabolism microenvironment plays an important role in the establishment and maintenance of SCs heterogeneity and revealed that MyoD as a metabolic sensor mediates tissue metabolism microenvironment to maintain Pax7Hi and Pax7Mi subpopulations in sarcopenia [49]. At present, the molecular mechanism by which SCs heterogeneity is established and maintained during development is unclear. Many studies have confirmed that a greater number of SCs are attached to the slow muscle than the fast muscle. The gene expression is also different, such as AChE [50], FGF receptor [51] and Pax3/Pax7 [52-54], resulting in significant differences in proliferation rates and differentiation capabilities [55, 56]. However, the role of microenvironmental metabolism of myofibers in SCs biology has not elucidated. Studies have evaluated the pattern of myoglobin expression after differentiation of SCs from slow or fast muscle into myotubes: SCs from avian and rodent fast muscles produce myotubes that express only MyHC-II, but SCs from slow muscle fibers produce myotubes expressing MyHC-I and MyHC-II [53, 54]. In addition, by inducing differentiation of the SCs of the tibialis anterior muscle (fast muscle) and the soleus muscle (slow muscle), it was found that the contractile ability of the formed myotubes was significantly different, indicating that SCs from fast and slow muscles produce different functional myofibers after differentiation in vitro [53]. Interestingly, unlike birds and rodents, almost all myotubes co-express MyHC-I and MyHC-II after differentiation of human fast and slow myofiber-derived SCs [57]. Therefore, it is necessary to further demonstrate the diversity of the source "fast muscle" and "slow muscle" SCs in vivo. In the study of skeletal muscle transplantation in rodents, it was found that the ability of muscle regeneration depends more on the age of the host, indicating that microenvironmental changes are one of the important factors for the decline of skeletal muscle SCs function [58]. The SCs from soleus were transplanted into the extensor digitorum long muscle, and slow-twitch muscle fibers were observed after 3 months [59]. However, this phenomenon was not observed after 6 months, suggesting that the skeletal muscle metabolic microenvironment can effectively reprogram mature regenerated myofibers, but not the SCs themselves. Many studies have found that changes in myofiber metabolism after stimulation by oxidative or glycolysis, respectively, indicating that SCs are malleable [59, 60]. The above studies confirmed that changes in skeletal muscle microenvironment caused a decrease in SCs content, which may be a key factor causing fast-twitch oxidized myofiber atrophy leading to sarcopenia.

## Signaling pathways and transcription factors that regulate skeletal muscle metabolism

**Figure 3.**
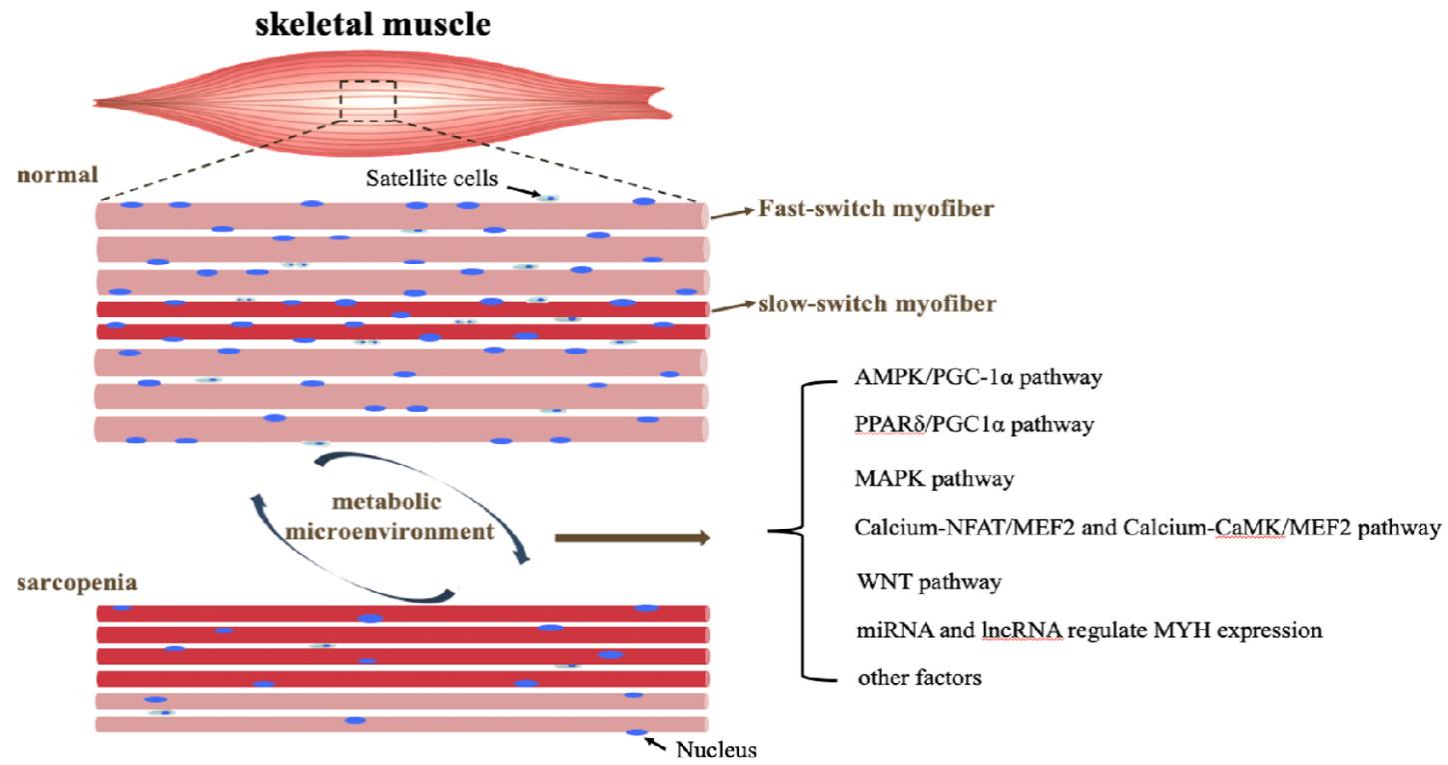
The model of signaling pathways and transcription factors that regulate metabolic microenvironment in skeletal muscle. AMPK/PGC-1α signaling, MAPK signaling, Calcium-NFAT/MEF2 and Calcium-CaMK/MEF2 signaling, WNT signaling, miRNA and lncRNA and other transcription factors.

The skeletal muscle metabolism microenvironment is highly plastic. The researchers found that some of the signaling pathways involved in intracellular energy metabolism and many transcription factors (Fig. 3), such as PGC-1α signaling, Calcium-NFAT/MEF2 and Calcium-CaMK/MEF2 signaling, MAPK signaling, WNT signaling, miRNA and lncRNA and other transcription factors. These factors may regulate the microenvironment of skeletal muscle metabolism, promote the differentiation of SCs and the transformation of myofiber metabolism to affect muscle function. Most intracellular signaling pathways involved in muscle homeostasis are affected in aging and could therefore be exploited as targets for the development of interventions aimed at preventing, delaying, or reversing sarcopenia.

## PGC-1α regulates skeletal muscle metabolism

Peroxisome proliferator-activated receptor-gamma coactivator-1 (PGC-1α) is an important factor required for mitochondrial biogenesis, skeletal muscle production and oxidative metabolism and oxidative myofiber formation [61, 62]. Exercise strongly induces PGC-1alpha expression in muscle, and overexpression of PGC-1alpha in skeletal muscle activates mitochondrial oxidative metabolism and neovascularization, leading to markedly increased endurance. In light of these findings, PGC-1alpha has been proposed to protect from age-associated sarcopenia and whole-body metabolic dysfunction [63, 64]. After overexpression of PGC-1α in the muscle creatine kinase (MCK) promoter, it was found that there was no significant effect on muscle mass in young mice, but it could prevent myasthenia in aged mice by inhibiting protein degradation [65]; however, after overexpression of PGC1α in the human skeletal actin (HSA) promoter, muscle atrophy is observed at 25 weeks of age in mice [66]. The model mice have “dark” muscle fibers, lose weight, increase energy expenditure, and aggravate glycolysis myofibers. MCK-PGC-1α drives PGC-1α protein level expression, similar to the endogenous physiological level present in slow myofibers; in contrast, HSA-PGC-1α transgenic mice show more than 10-fold PGC-1α protein expression levels, therefore, physiological levels of PGC-1α protect fibers from atrophy, but excessive levels of PGC-1α will cause muscle atrophy, especially for glycolysis fibers. Though the mechanism by which PGC-1α is expressed in different promoters on muscle mass and function is still unknown, we still know that PGC-1α plays an important role in the regulation of skeletal muscle metabolism and skeletal muscle atrophy is a positive effector of oxidative myofiber development [67, 68].

### 1) AMPK/PGC-1α signaling

Activated 5'-AMP-protein kinase (AMPK) in skeletal muscle continues to reduce the free [ATP] / [ADP] ratio to an important signal that initiates change in myofiber metabolism types [76], at the same time increase glucose uptake, fatty acid oxidation, glycogen metabolism, protein synthesis and mitochondrial biological functions. Rockl et al. demonstrated that AMPKα2 activity was reduced in skeletal muscle of aging or sarcopenia patients, and skeletal muscle metabolic conversion was reduced, leading to skeletal muscle atrophy. During endurance training, AMPK activation in skeletal muscle promoted glycolysis myofibers to fast-twitch oxidized myofiber transition [69-71]. AMPK controls the expression of PGC-1α by phosphorylating HDAC5, and transcriptional activation of PGC-1α promotes release of the enhancer MEF2, a histone deacetylase inhibitor [72, 73]. *In vivo* studies have shown that MEF2 is involved in the regulation of the expression of oxidized myofiber genes [74]. PGC-1α also activates the calcium neuro-phosphatase pathway and upregulates the transcription of MEF2 protein [75]. Overexpression of AMPK in mouse skeletal muscle results in the conversion of glycolytic myofibers into oxidized myofibers [76, 77]. 5-aminoimidazole-4-carboxamide-nucleoside (AICAR) is used as an AMPK activator. Some studies have found that long-term administration of AICAR promotes mitochondrial proliferation and induces the conversion of glycolysis to oxidized fiber during endurance exercise and oxidative training [78, 79]. Recent studies have found that activated AMPK regulates follicle action protein 1 (FNIP1) alters the type of myofiber metabolism [80]. Deletion of FNIP1 leads to increased activation of AMPK in skeletal muscle and promotes the conversion of glycolytic myofibers to PGC-1α-dependent oxidized myofibers [81, 82]. However, the role of AMPK in mediating the conversion of glycolysis to oxidized myofibers remains unclear. AMPKγ3 transgenic mice and AMPKβ1β2 knockout mice showed normal fiber distribution, while the percentage of slow-twitch oxidized myofibers was found to increase in muscle-specific knockout of AMPKα1α2 [83]. Taken together, these data indicate that AMPK induces skeletal muscle to oxidized myofibers via PGC-1α.

### 2) PPARδ/PGC-1α signal

The attention given to PPARs has also gained importance due to their role in muscle pathophysiology associated with the metabolic syndrome, myopathies, muscular dystrophies and sarcopenia [84, 85]. Coincidentally, the incidence of sarcopenia (age-related muscle loss) has been shown to correlate with reduced levels of PPARδ, and pharmacological activation of PPARδ has been shown to reduce the incidence of sarcopenia by increasing nuclear accretion in myofibers [86]. Alison R Angione et al found that Myf5-Cre/PPARδ flox/flox mice had 40% fewer SCs than their wild-type littermates, and these SCs exhibited reduced growth kinetics and proliferation in vitro. Furthermore, regeneration of Myf5-Cre/PPARδ flox/flox muscles was impaired after cardiotoxin-induced injury [87]. Previously, genomic studies have found that calcineurin, calmodulin-dependent kinase, PGC-1α and activated PPARδ form a signaling network that regulates skeletal muscle metabolism, protecting insulin resistance and obesity. The latest data by Wang et al [88] revealed that PPARδ-mediated transcriptional pathways are involved in the regulation of skeletal muscle metabolic phenotypes. PPARδ transgenic mice showed an increase in the proportion of oxidase, mitochondria and oxidized myofibers; on the contrary, oxidized myofibers in PPARδ knockout mice decreased [76], while PGC1α expression in skeletal muscle also decreased, indicating that activated PPARδ plays a key role in oxidative myofiber development. Importantly, skeletal muscle metabolism type regulation in transgenic animals is the result of altered mRNA expression during muscle development, and whether a similar process involves changes in myofiber metabolism in mature muscle remains to be determined. Interestingly, the activation of PPARβ/δ ameliorates the human Duchenne muscular dystrophy (DMD) phenotype in X-linked muscular dystrophy (mdx) mice that have a spontaneous mutation in the dystrophin gene. PPARβ/δ regulates Utrophin A by directly binding to the PPRE in the Utrophin A promoter region [89]. PPARδ/PGC1α-dependent signaling plays an important role in the induction and maintenance of oxidized myofibers, other modulators, including calcineurin are also involved [90]. The calcineurin/NFAT signaling-dependent activation of Utrophin A also ameliorates the DMD phenotype in mdx mice [91]; however, pharmacological inhibition of cyclosporin A [92] on calcineurin resulted in inhibition of PPARδ-mediated changes in myofiber metabolism, indicating cross-interference between these different pathways. Recent studies have found that PGC1α activates HIF-2α through ERRα transcription, and that increased oxidized myofibers are found in HIF-2α knockout mice [93]; In addition, HIF-2α promotes calmodulin activation by binding to calcium, resulting in an increase in calcium-regulated phosphatase A activity, driving the regulation of slow oxidized myofiber gene expression. PGC1α synergizes with calcineurin/nuclear factor of activated T cells (NFAT) signaling pathway to regulate oxidative myofiber formation, which serves as an essential pathway for maintaining oxidative myifiber phenotype [94].

## Calcium-NFAT/MEF2 and Calcium-CaMK/MEF2 signals

Calcineurin/NFAT signal is a key signal pathway to balance protein synthesis and protein degradation system, and it is necessary to maintain muscle mass. The calcineurin/NFAT signaling pathway regulates the transcriptional activity by promoting a slow genetic program and consequently would promote the transcription of genes encoding certain muscle proteins [95]. Using electrical stimulation to activate the calcineurin signal pathway can promote neuromuscular fiber remodeling and improve sarcopenia in the sedentary elderly [96]. In addition, Calcineurin is a Ca^2+/^cadherin phosphorylation-activated skeletal muscle-dependent myofiber metabolism [97]. Calcineurin and NAFT are involved in the conversion of glycolytic myofibers to oxidized myofibers [98]. Activated calcineurin dephosphorylates NFAT and then translocates into the nucleus to induce oxidative myofibrillar-specific gene transcription [74, 98]. Activated calcineurin also dephosphorylates nuclear myocyte enhancer factor 2 (MEF2) [74], and *in vivo* studies have shown that MEF2 is involved in the regulation of oxidized myofiber gene expression [74]; MEF2 is also associated with myogenic alkaline helix - Loop-helix (bHLH) muscle regulatory factor MyoD interaction and regulate transcription of myogenic genes [99]. Meissner et al. used calcium ionophore to induce C2C12 differentiation, and myotubes showed a complex of NFATc1, MyoD, MEF2D and coactivator p300 to upregulate the slow myosin promoter in a manner dependent on calcineurin [100]. The Ca^2+^/calmodulin-dependent kinase (CaMK) pathway is also a major signal transduction pathway involved in the conversion of glycolytic-type myofibers to oxidized myofibers [101, 102]. Calcium-activated CaMK phosphorylates members of the type II histone deacetylase protein (HDAC) family [103]. Type II HDAC dephosphorylates the nuclear MEF2 and loses its activity, so activated CaMK promotes increased MEF2 transcriptional activity. Although these two signaling pathways (Ca^2+^/NFAT and Ca^2+^/CaMK) play a role in the transformation of myofiber metabolism types, the interrelationship and molecular mechanism between them remain unclear. For example, Sworp et al. injected activated calcineurin or NFAT2 expression plasmids into adult myofibers that do not activate gene expression of the slow myosin light chain [90].

## MAPK signal

The mitogen-activated protein kinase (MAPK) protein family in skeletal muscle is composed of four distinct signaling modules: 1) extracellular signal-regulated kinase ERK1/2 (ERK1/2); 2) p38 MAPK; 3) c-Jun NH 2 - terminal kinase (JNK); and 4) ERK5 [104, 105]. MAPK is activated by phosphorylation of threonine and tyrosine residues of MAP kinase and is inactivated by dephosphorylation of specific phosphatase. MAPK phosphatase selectively dephosphorylates and inactivates nuclear phosphatase of JNK, ERK1/2 and p38 MAPK, and knockdown of MAPK results in activation of JNK, ERK2 and p38 in skeletal muscle [106]. Activation of the ERK1/2, p38, and JNK signaling cascades has been extensively studied in skeletal muscle contraction. Endurance training causes MAPK signaling cascade activation to regulate skeletal muscle metabolism [107]. It has been reported that the upstream kinase of p38 activates MyoD to promote the increase of fast-twitch oxidized myofibers; Meissner also reported that p38 controls glycolytic (IIx) promoter activity in myotubes [106]. Recent studies have found that ERK1/2 activity is more than twice as high in glycolytic myofibers than in oxidized myofibers, suggesting that the ERK1/2 pathway may play an important role in the glycolytic myofiber phenotype. The Higginson group [108] showed that inhibition of the ERK1/2 pathway in rat and mouse myocytes reduced glycolytic myofibers and increased oxidized myofibers. In addition, the Ras/MEK/ERK-linked motor neuron signaling system plays a key role in the regeneration of mouse soleus muscle oxidized myofibers [109]. Joachim demonstrated that phosphorylation of ERK1/2 activates the phosphorylation site on p300 serine and then acetylated NFATc1, which enhances MyHC Iβ transcript levels and promotes muscle to oxidative transformation [106]. Boyer JG‘s results also suggest sustained MEK1-ERK1/2 activity in skeletal muscle produces a fast-to-slow fiber-type switch that protects from muscular dystrophy [110].

## WNT signal

The WNT signaling pathway also plays an important regulatory role in skeletal muscle metabolism [111]. The WNT signaling pathway mainly includes three signaling pathways: 1) the classical WNT signaling pathway dependent on β-catenin, which is transferred to the nucleus by the stable expression of intracellular β-catenin and binds to the LEF/TCF transcription factor to regulate downstream. Target gene; 2) non-classical WNT signaling pathway independent of β-catenin, Wnt/Ca2+ signaling pathway, and 3) cell-plane polar pathway Wnt/polarity signaling pathway [112]. According to the different biological functions of β-catenin dependence, the WNT family is divided into two categories: one, dependent on β-catenin and activates the canonical Wnt signaling pathway, including: WNT1, WNT2, WNT3, WNT3a, WNT8, WNT8b and WNT10b;Second, it does not rely on β-catenin to activate non-canonical signaling pathways, including: WNT2b, WNT4, WNT5a, WNT5b, WNT6, WNT7a, WNT7b, WNT9a, WNT9b, WNT10a, WNT11 and WNT16. In recent years, studies have shown that Wnt family members Wnt1 [113] and Wnt3a [114] promote skeletal muscle SCs differentiation and regulate the expression of Myod and Myogenin through Wnt/β-catenin signaling pathway, and studies have confirmed that Myod is high expression in fast muscle, while Myogenin is expressed more in slow muscle. After knocking out β-catenin in mouse embryos, David et al found that oxidized myofibers in the limbs were significantly reduced. In addition, Kelly et al. used MyHC-specific antibodies to detect the effect of WNT signaling on myocyte differentiation in vitro, and found that Wnt5a [115] Promotes an increase in slow-oxidation myofibers; Wnt11 [116] is the opposite. Myostatin is involved in early muscle formation, activates Myostatin expression, and preferentially reduces oxidized myofibers; however, knocking out Myostatin in mice, found that Wnt/β-catenin signaling is down-regulated, and regulation of slow oxidized myofiber-related gene expression is reduced [99]. Tee et al. demonstrated in zebrafish embryos that the Wnt/β-catenin classical signaling pathway interacts with myostatin to cause a slow-oxidation muscle increase. It is speculated that myostatin regulates the development of slow-oxidation myofibers through the Wnt/β-catenin signaling pathway. When the Axin1/APC1 mutation is found in the non-canonical WNT signaling pathway in zebrafish, the glycolysis-type myofibers are degraded [117]. The above data show that the classical Wnt signaling pathway not only promotes myogenic differentiation *in vivo*, but also plays an important regulatory role in the composition of skeletal muscle metabolism types.

## miRNA and lncRNA regulate MYH expression

miRNAs are classes of conserved non-transcribed RNAs that regulate protein expression after transcription. miRNA-208a, -208b and -499 are each encoded by an intron of the different myosin heavy chain genes MYH6, MYH7 and MYH7b, respectively [118-120]. In particular, miR-208b [121] and miR-499 [119] upregulate the slow muscle gene and inhibit fast muscle gene expression by activating Sox6 and Purb expression; in contrast, miR-1 and miRNA-133 are mainly expressed in fast muscle [118]. Zhang et al used thyroid hormone receptor knockout model mice to confirm that miRNA-133a targets TEA domain family member 1 (TEAD1) to regulate oxidized muscle to glycolytic muscle transformation [15]. In addition, recent studies have found that miRNA-378 regulates mitochondrial metabolism and bioenergy metabolism by regulating PGC1-β, including gluconeogenesis, glycolysis, and fatty acid oxidation, ultimately regulating the type of myofiber metabolism; inhibiting miRNA-378, up-regulating MyoR and PGC -1β expression leads to increased mitochondria, capillary density, and oxidized myofibers [122].

lncRNA is a new class of RNA that is greater than 200 nucleotides but does not encode a protein. Several lncRNAs have been identified in different species related to skeletal muscle development, disease, and skeletal muscle metabolism [123, 124]. Recent evidence suggests that a skeletal muscle-specific lncRNA-linc-MD [125] inhibits miRNA133 and regulates MAML1 and MEF2C expression. Interestingly, in the muscle cells of patients with Duchenne muscular dystrophy, the level of linc-MD1 was significantly reduced, and linc-MD1 was found to promote the increase of fast muscle, but the molecular mechanism is still unclear. Han [126] and colleagues report that the lncRNA transcript from the antisense strand of the MYH7 locus regulates the balance between α-MHC and β-MHC expression by modulating the Brg1-HDAC-PARP chromatin complex as a molecular switch. By identifying alternative splicing antisense transcripts of the MYH7 gene, the authors found that these lncRNAs are the splicing isoforms of Mhrt and are closely related to the ratio of the gene subtype MYH6/MYH7.

## Other transcription factors

The Six1/Eya1 complex composed of members of the Six family is involved in skeletal muscle reprogramming of oxidized myofibers to glycolysis myofibers [127]. Follicle protein interacting protein-1 (Fnip1) [81] linked AMPK to PGC1α signaling to promote differentiation of myoblasts into slow oxidized myofibers. Prospero related homeobox1 (Prox1) [128] transcription factor activates calcineurin kinase signaling in C2C12 cells to induce slow oxidative myofibrillin expression. In contrast, the Foxhead transcription factor family member FoxO1 [129] inhibits calcineurin kinase signaling and promotes the conversion of slow-oxidized myofibers to rapidly oxidized myofibers. T-box 15 (Tbx15) [130] transcription factor activates AMPK signaling, reduces IGF2 expression, promotes protein expression of glycolytic muscle fibers, and plays an important role in muscle metabolism.

## Conclusions

Sarcopenia is an age-associated condition which links to multiple etiological factors ranging from external factors (physical activities, nutrients, and diseases) to internal factors (interplay between different cells, regulation of signal pathways, and congenital genetic configuration of an individual). Metabolic microenvironment changes within the skeletal muscle with aging, and these changes are associated in part with age-related skeletal muscle remodeling. Current research has demonstrated that the skeletal muscle metabolic microenvironment can directly affect muscle function and regeneration in response to numerous physiological stressors, such as changes in physical activity, energy utilization, and even aging. The studies presented in the current discussion have identified numerous ways in which metabolism can directly influence protein synthesis and transcription. It is in this manner that metabolic remodeling can play a large role in both physiologic and pathologic adaptations during a disruption in homeostasis. In this article, we propose that the differences in skeletal muscle metabolism may determine the different responses of distinct types of myofibers to skeletal muscle aging signals. Reprogramming skeletal muscle energy metabolism may interfere with the development of sarcopenia. The different responses of different types of myofibers to skeletal muscle aging signals provide some possible clues for studying the pathogenesis of sarcopenia. Most intracellular signaling pathways involved in intramuscular metabolism and homeostasis are affected and can be used as targets to explore interventions aimed at preventing, delaying or reversing sarcopenia.
